# Supplementary material for: Polymersomes preventing brain infiltration of CD177+ neutrophils to mitigate hemorrhagic transformation post-tPA thrombolysis
Source: Nat Commun. 2026 Mar 25;17:4395. doi: 10.1038/s41467-026-71076-w (PMC13181025; doi:10.1038/s41467-026-71076-w)
Supplement: Supplementary file 1 — Supplementary Information [file 41467_2026_71076_MOESM1_ESM.pdf]

Supplementary Information

Polymersomes preventing brain infiltration of CD177$^+$ neutrophils to mitigate hemorrhagic transformation post-tPA thrombolysis

Zhenhua Wang[1], Hua Liu[2], Zhiyao Xu[2], Cheng Huang[1], Xing Guo[1,*], and Shaobing Zhou[1]

[1]Institute of Biomedical Engineering, College of Medicine, Southwest Jiaotong University, Chengdu 610031, P. R. China.

[2]Department of neurology, the third people's hospital of Chengdu, Chengdu 610031, PR China

*Corresponding author

Email: xingguo@swjtu.edu.cn

1. Supplementary Figures

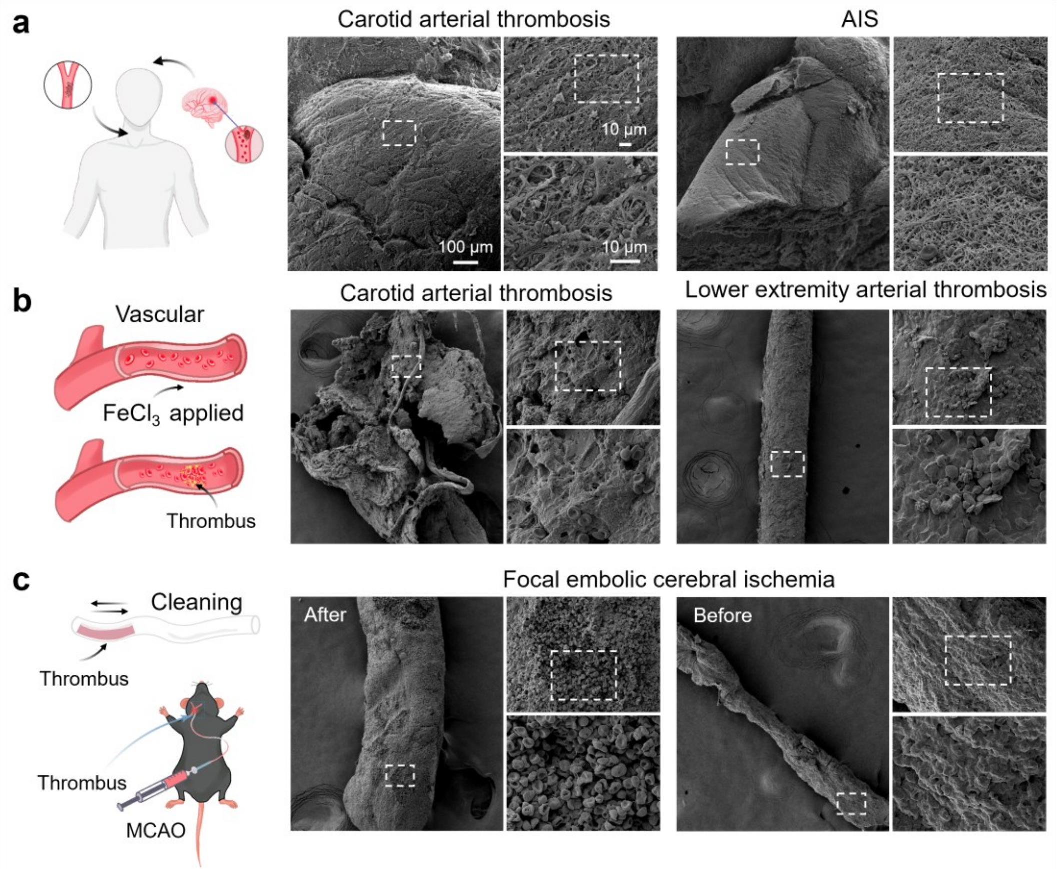

Supplementary Fig. 1. Scanning electron microscope (SEM) images of different types of thrombosis. Schematic illustrations of thrombosis and SEM images from patients with total carotid embolism (left) and cerebral middle artery embolism (right) (**a**), carotid artery thrombosis model (left) and lower extremity arterial thrombosis (right) induced by $FeCl_3$ (**b**), and the mouse embolic middle cerebral artery occlusion model (**c**). The results in (**a, b, c**) were representative of three independent experiments.

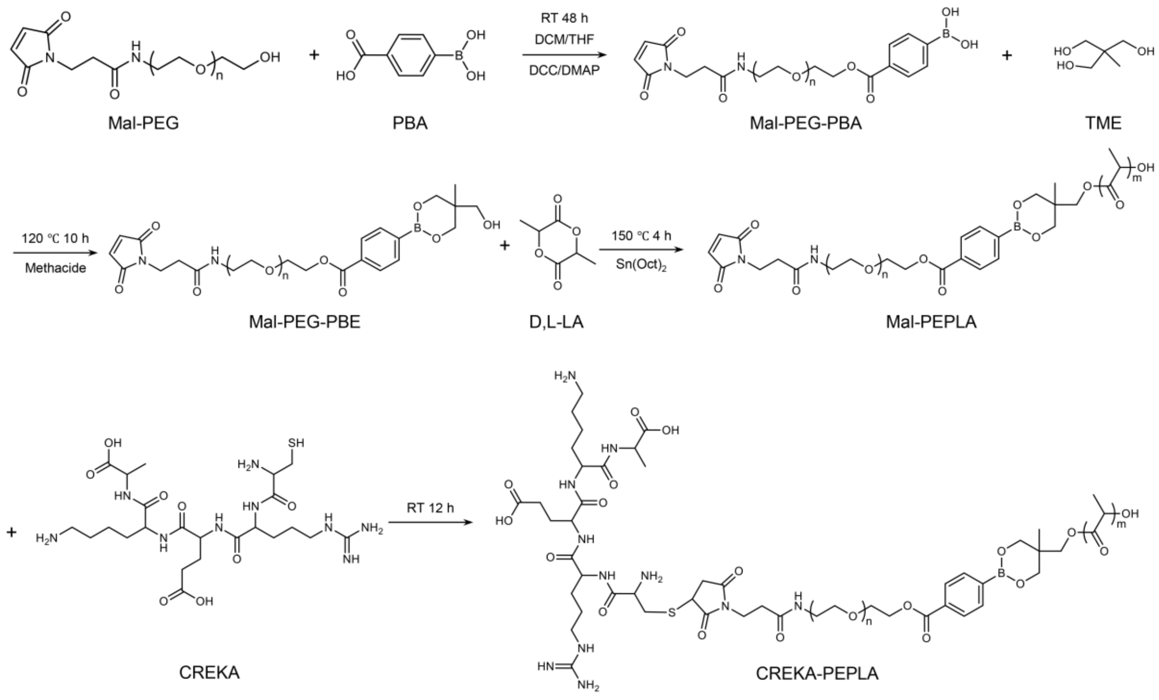

Supplementary Fig. 2. Synthesis route of CREKA-PEPLA polymer.

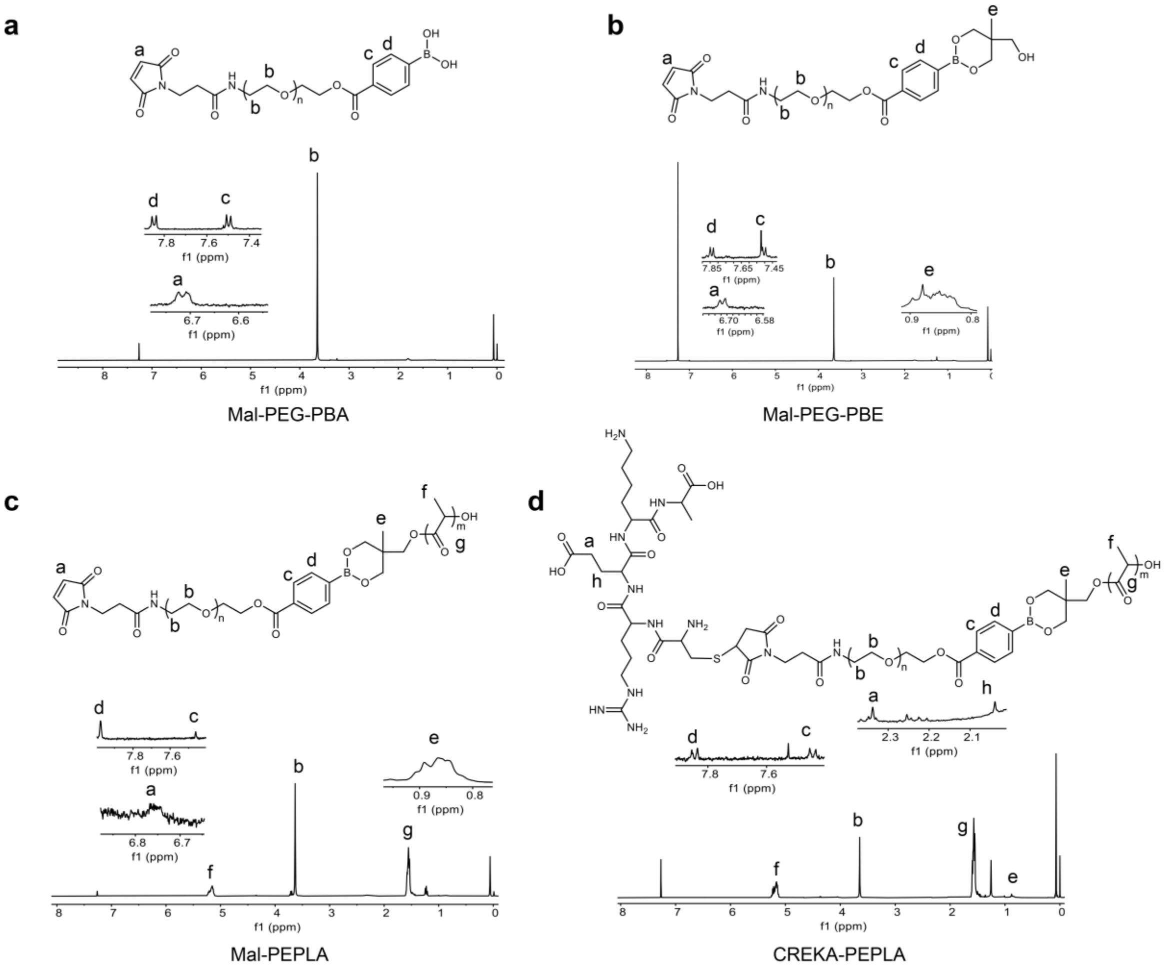

Supplementary Fig. 3. ¹H NMR spectra of intermediates and final product. ¹H NMR spectra of Mal-PEG-PBA (**a**), Mal-PEG-PBE (**b**), Mal-PEPLA (**c**), and CREKA-PEPLA (**d**) in $CDCl_3$.

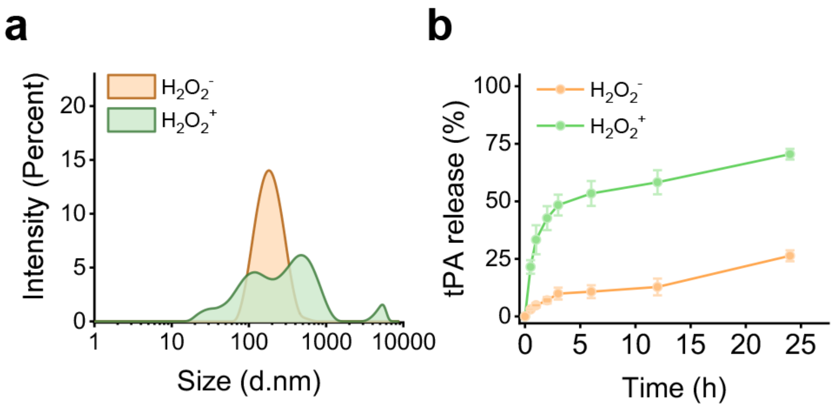

Supplementary Fig. 4. Characterization of responsive properties of P polymersomes. a, Size distribution of P polymersomes in phosphate buffered saline (PBS) or H_2O_2. **b,** In vitro tPA release from P polymersomes in PBS or H_2O_2 (n = 3 biologically independent samples). Data in (**b**) were presented as mean ± SD. Source data are provided as a Source Data file.

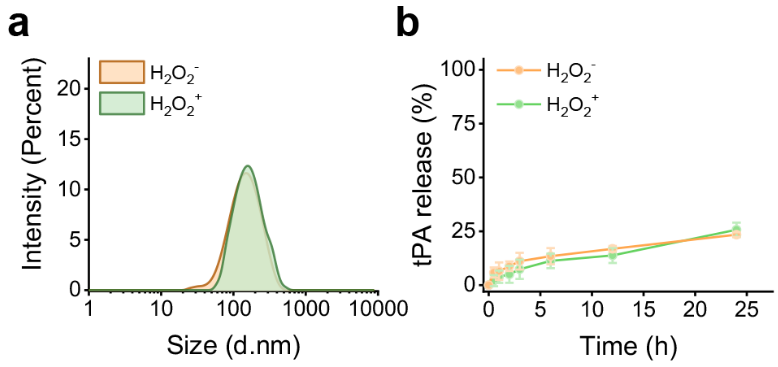

Supplementary Fig. 5. Characterization of responsive properties of Null polymersomes. a, Size distribution of Null polymersomes in PBS or H_2O_2. **b,** *In vitro* tPA release from Null polymersomes in PBS or H_2O_2 (n = 3 biologically independent samples). Data in (**b**) were presented as mean ± SD. Source data are provided as a Source Data file.

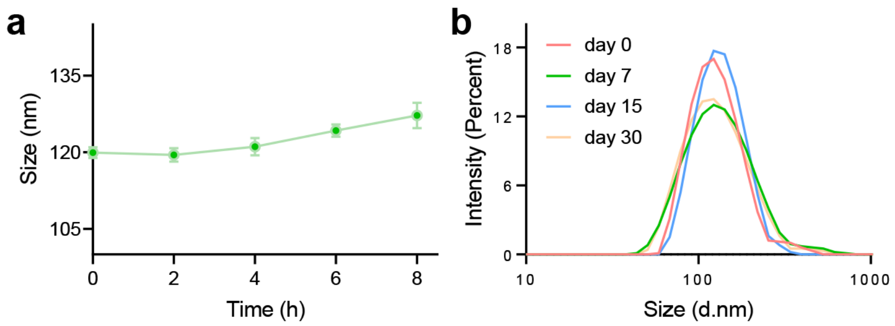

Supplementary Fig. 6. Stability of CP@tPA. a, Colloidal stability of CP@tPA incubated in fetal bovine serum (FBS) (pH 7.4) within 8 h at 37 ° C (n = 3 biologically independent samples). **b,** Size distribution of CP@tPA in PBS for 30 days. Data in (**a**) were presented as mean ± SD. Source data are provided as a Source Data file.

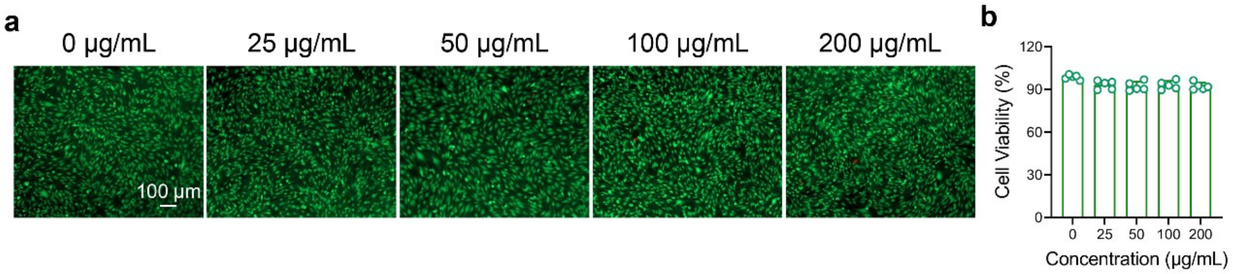

Supplementary Fig. 7. In vitro cytocompatibility of CP@tPA polymersomes. Live/dead staining (**a**) and cell viability (**b**) showing in vitro cytotoxicity of bEnd.3 treated with different concentrations of CP@tPA polymersomes for 24 h (n = 5 biologically independent samples). Data in (**b**) were presented as mean ± SD. Source data are provided as a Source Data file.

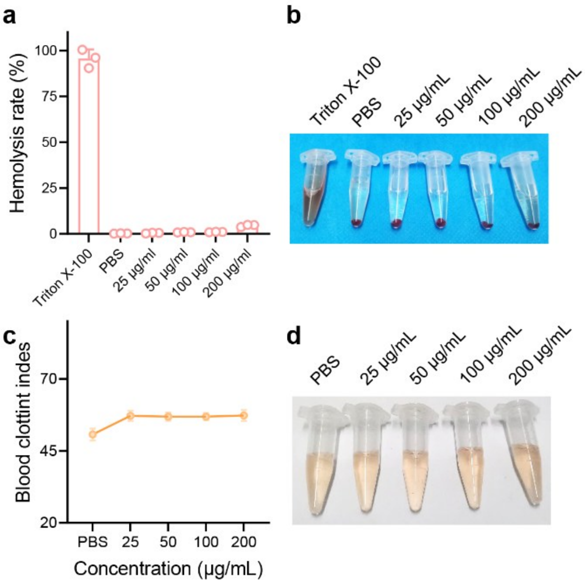

Supplementary Fig. 8. Evaluation of hemocompatibility of CP@tPA polymersomes. Hemolysis (**a,b**) and blood coagulation (**c,d**) evaluations of CP@tPA polymersomes at different concentrations. Data in (**a**, **c**) were presented as mean ± SD, *n* = 3 biologically independent samples. Source data are provided as a Source Data file.

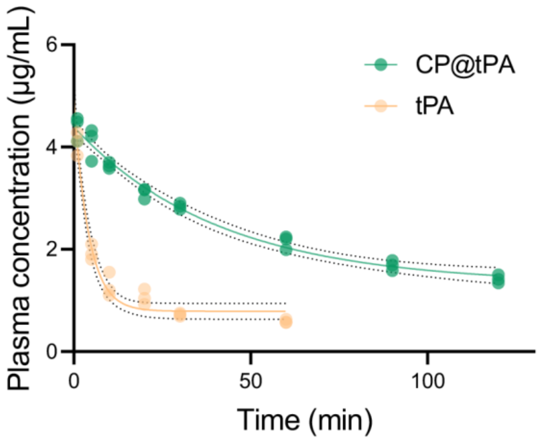

Supplementary Fig. 9. Pharmacokinetic profiles of CP@tPA and tPA (*n* = 3 biologically independent samples). Source data are provided as a Source Data file.

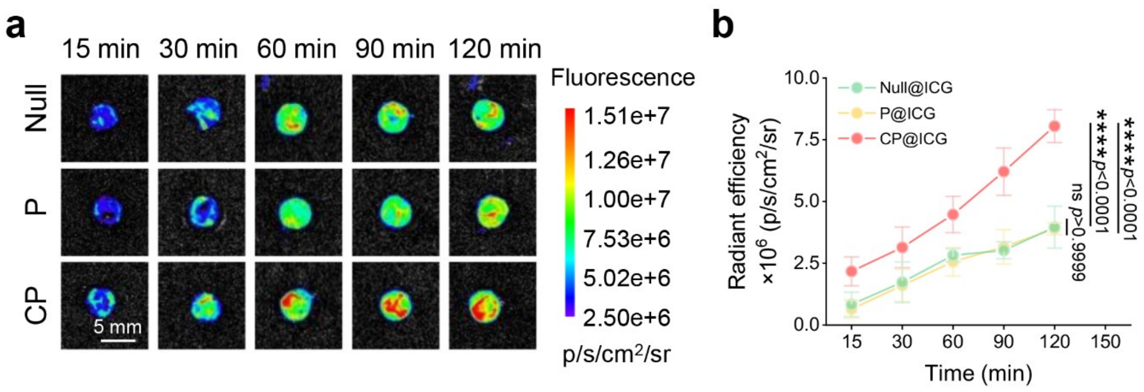

Supplementary Fig. 10. In vitro thrombus targeting of different polymersomes. a, Fluorescence images of ICG-loaded polymersomes in the *in vitro* thrombus. **b,** The average radiation efficiency of ICG-loaded polymersomes in the thrombus based on fluorescence intensity (n = 3 biologically independent samples). Data are presented as the mean ± SD. P values were calculated by two-way ANOVA followed by Bonferroni's post-hoc test. Source data are provided as a Source Data file.

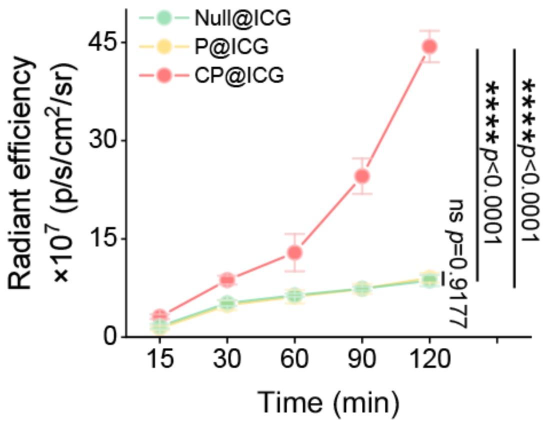

Supplementary Fig. 11. The average radiation efficiency of ICG-loaded polymersomes in the mice brains based on fluorescence intensity (n = 3 biologically independent samples). Data are

presented as the mean ± SD. *P* values were calculated by two-way ANOVA followed by Bonferroni's post-hoc test. Source data are provided as a Source Data file.

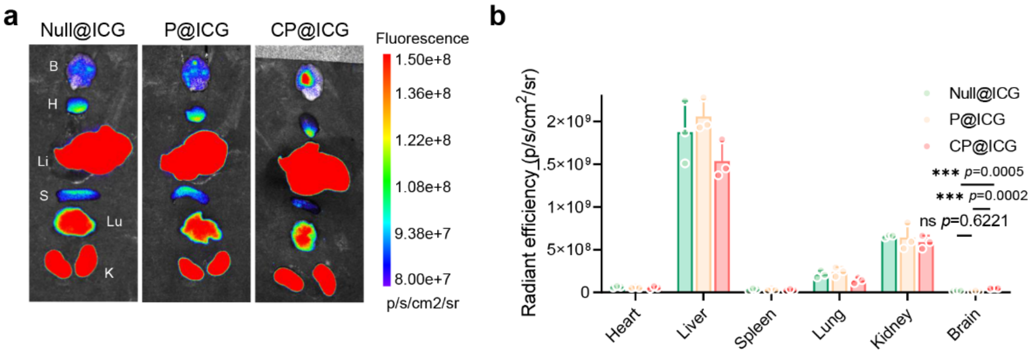

Supplementary Fig. 12. Tissue distribution of different polymeric vesicles. a, Ex vivo fluorescence imaging showing ICG-loaded polymersomes in mouse tissues at 2 h post-injection. B, brain; H, heart; Li, liver; S, spleen; Lu, lung; K, kidney. **b,** The average radiation efficiency of ICG-loaded polymersomes in the mice tissues based on fluorescence intensity (*n* = 3 biologically independent samples). Data are presented as the mean ± SD. *P* values were calculated by One-way ANOVA followed by Bonferroni's post-hoc test. Source data are provided as a Source Data file.

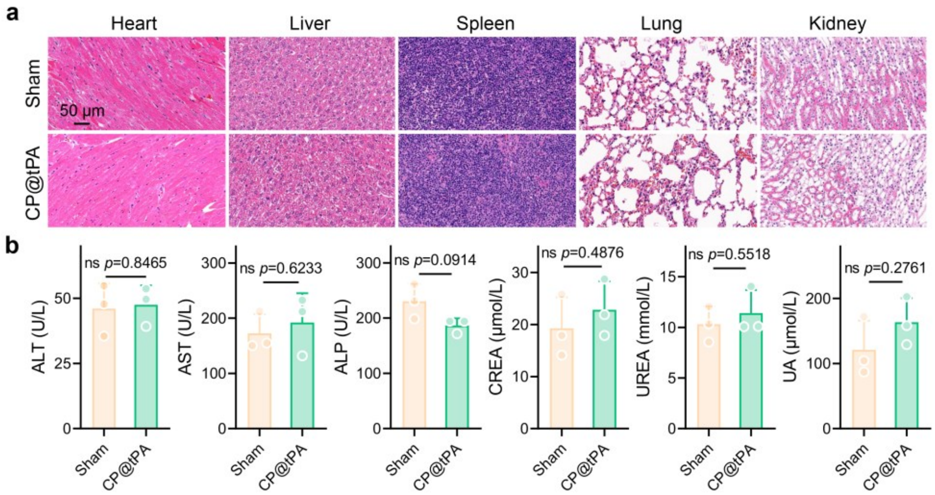

Supplementary Fig. 13. Evaluation of biocompatibility and biosafety. H&E staining of heart, liver, spleen, lung, and kidney (**a**) and serum biochemical indices (**b**) in sham and CP@tPA groups 24 h poststroke (n = 3 biologically independent samples). The results in (**a**) were representative of three independent experiments. Data are presented as the mean ± SD. P values were calculated by two-tailed unpaired t-test. Source data are provided as a Source Data file.

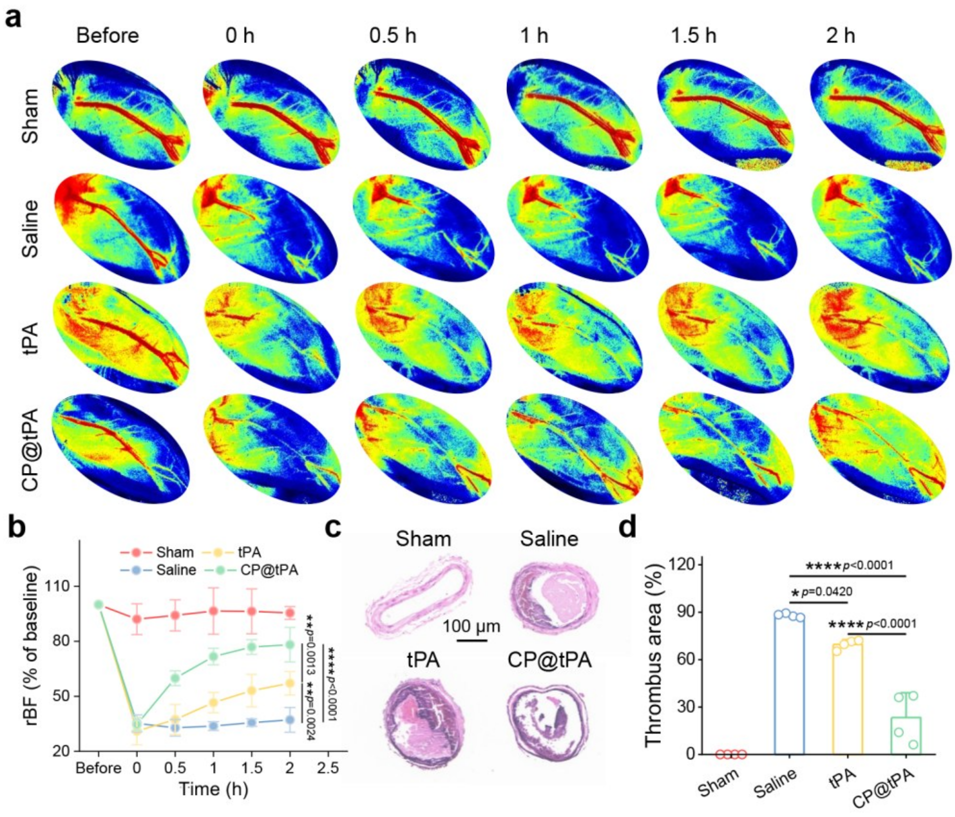

Supplementary Fig. 14. Evaluation of thrombolytic performance in the lower extremity arterial thrombus model. Laser speckle contrast images (**a**) and regional blood flow (rBF) quantification (**b**) of lower extremity arterial thrombus model in different treatment groups (*n* = 3 biologically independent samples). **c**) H&E staining of lower extremity arterial thrombus model in different treatment groups. **d**) The thrombus area was calculated by H&E staining of lower extremity arterial thrombosis model in different treatment groups (*n* = 4 biologically independent samples). Data are presented as the mean ± SD. *P* values were calculated by one-way ANOVA or two-way ANOVA followed by Bonferroni's post-hoc test. Source data are provided as a Source Data file.

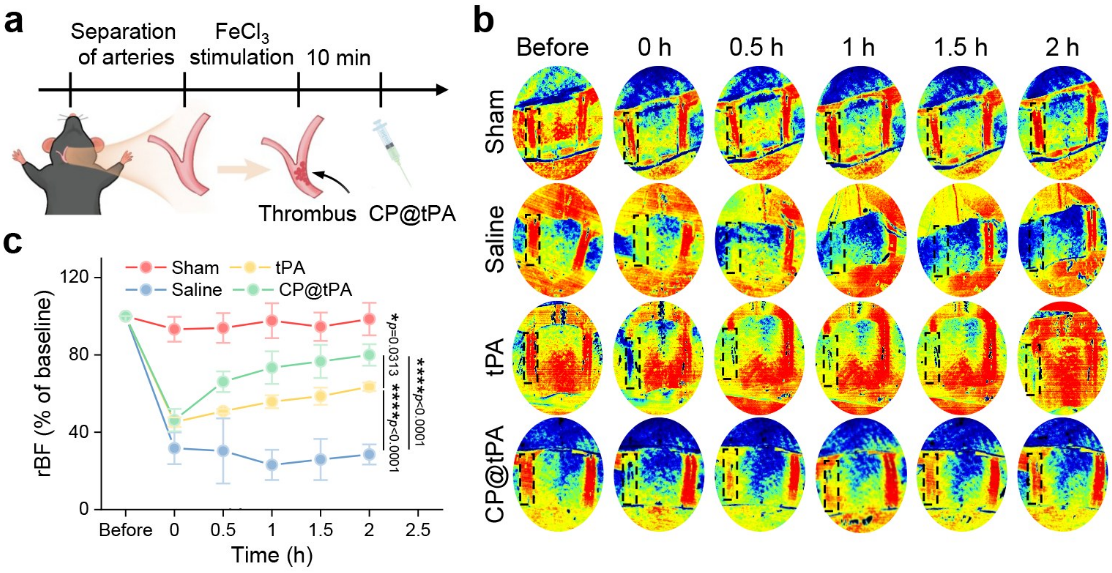

Supplementary Fig. 15. Evaluation of thrombolytic performance in the carotid artery thrombus model. a, Schematic illustration of carotid artery thrombus model construction. Laser speckle contrast images (**b**) and rBF quantification (**c**) of carotid artery thrombus model in different treatment groups (n = 3 biologically independent samples). Black boxes indicate the ischemic regions. Data are presented as the mean ± SD. P values were calculated by two-way ANOVA followed by Bonferroni's post-hoc test. Source data are provided as a Source Data file.

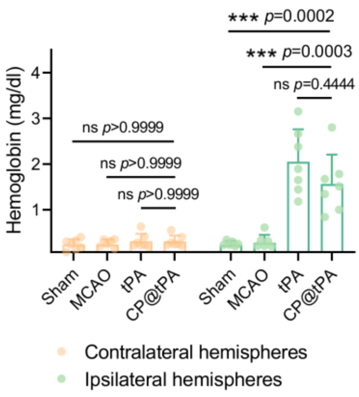

Supplementary Fig. 16. Hemoglobin content in the contralateral and ipsilateral hemispheres of mice in different treatment groups (n = 7 biologically independent samples). Data are presented as the mean ± SD. P values were calculated by one-way ANOVA followed by Bonferroni's post-hoc test. Source data are provided as a Source Data file.

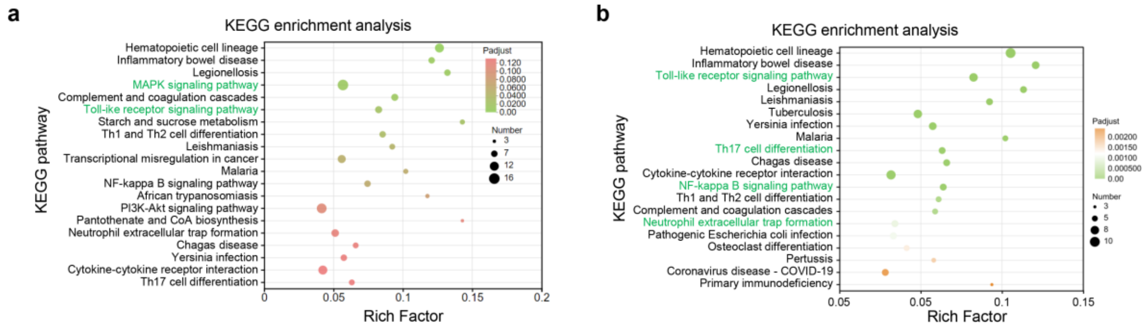

Supplementary Fig. 17. a, KEGG enrichment analysis of the differential genetic pathways between Stroke and hemorrhagic transformation (HT) groups. **b**, KEGG enrichment analysis of the differentially signaling related to immune system between Stroke and HT groups. Analyzed using a two-sided Fisher's exact test with Benjamini–Hochberg correction for multiple comparisons.

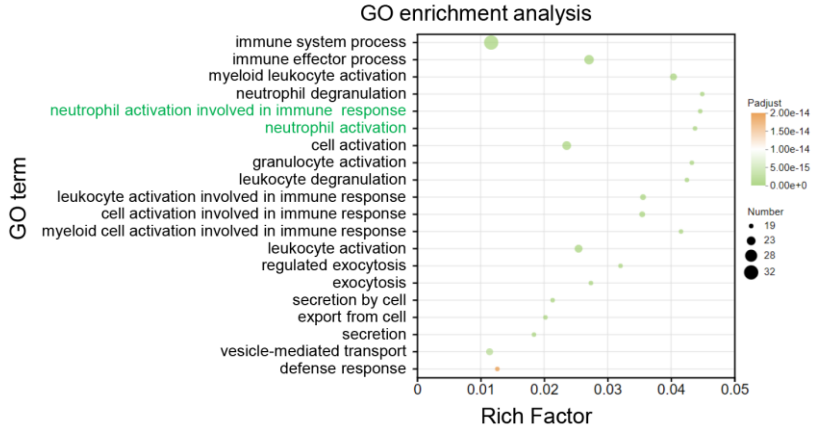

Supplementary Fig. 18. GO enrichment analysis of the differentially signaling related to innate immune system between Stroke and HT groups. Analyzed using a two-sided Fisher's exact test with Benjamini–Hochberg correction for multiple comparisons.

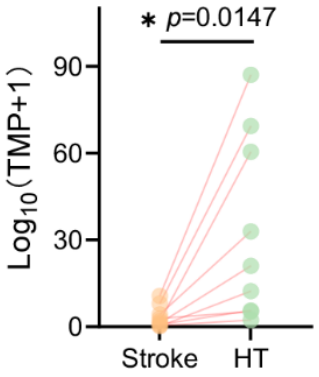

Supplementary Fig. 19. Transcriptomic analysis of *CD177* gene expression between Stroke and HT groups (n = 9 biologically independent samples). Data are presented as the mean ± SD. P values were calculated by two-tailed paired t-test. Source data are provided as a Source Data file.

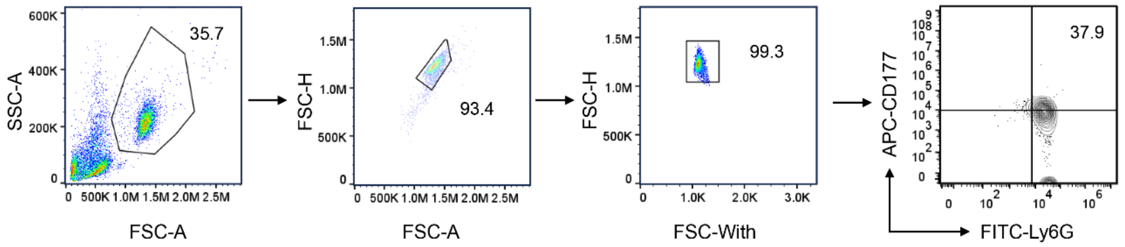

Supplementary Fig. 20. Flow cytometry gating strategy for identifying CD177^{+}Ly6G^{+} cells in mouse blood.

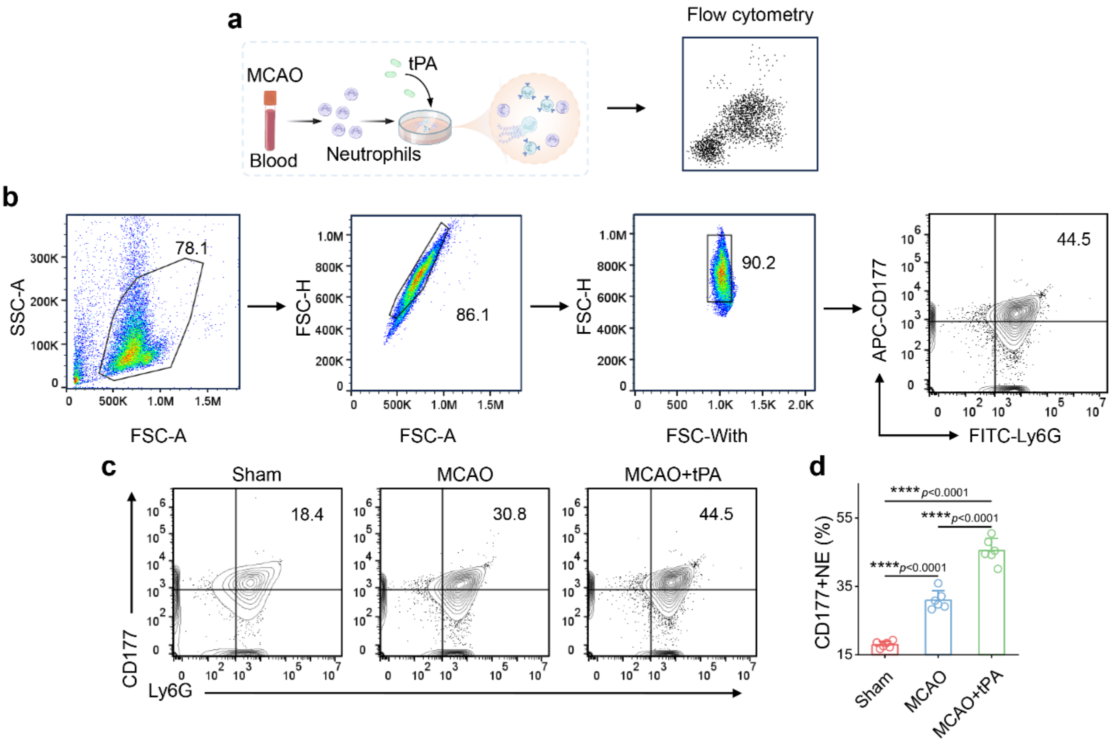

Supplementary Fig.21. Expression of CD177⁺ neutrophils after tPA stimulation. a, Schematic illustration of tPA-induced CD177⁺ neutrophil expression in vitro. Neutrophils isolated from the blood of middle cerebral artery occlusion (MCAO) mice 2 h poststroke were stimulated with tPA for 90 min to up-regulate CD177 expression. **b**, Flow cytometry gating strategy for identifying $CD177^{+}Ly6G^{+}$ cells in neutrophils. **c,** Representative flow cytometry plot of $CD177^{+}Ly6G^{+}$ cells neutrophils after tPA stimulation. **d,** Percentages of $CD177^{+}Ly6G^{+}$ cells according to flow cytometry analysis (n = 8 biologically independent samples). Data are presented as the mean ± SD. P values were calculated by one-way ANOVA followed by Bonferroni's post-hoc test. Source data are provided as a Source Data file.

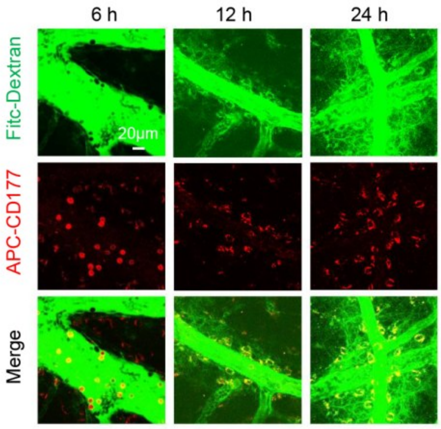

Supplementary Fig. 22. In vivo two-photon imaging showing the exacerbated vascular leakage by infiltrated CD177$^+$ neutrophils. Green, FITC-dextran (MW = 40 KDa); red, APC-CD177 labeled CD177$^+$ neutrophils. The results were representative of three independent experiments.

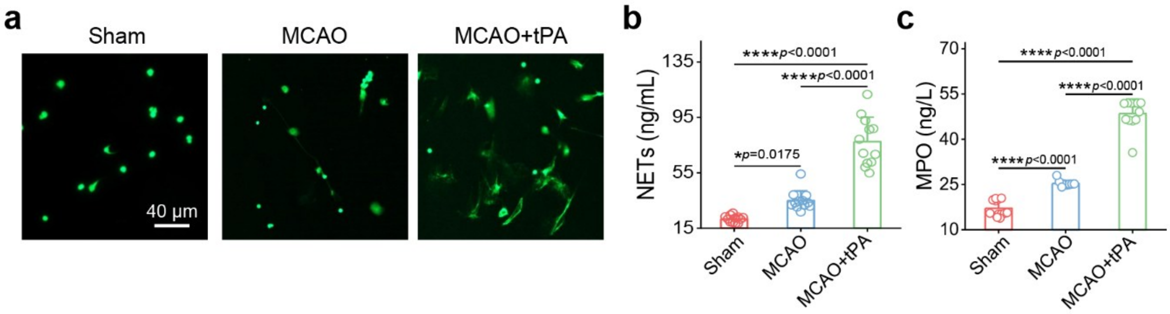

Supplementary Fig. 23. Expression of neutrophil extracellular traps (NETs) after tPA stimulation. a, Representative fluorescence images showing NETs (SYTOX$^+$, green) generation from neutrophils after 90 min of tPA stimulation. **b,c,** The NETs and myeloperoxidase (MPO) levels measured by ELISA (*n* = 12 biologically independent samples). Data are presented as the mean ± SD. *P* values were calculated by one-way ANOVA followed by Bonferroni's post-hoc test. Source data are provided as a Source Data file.

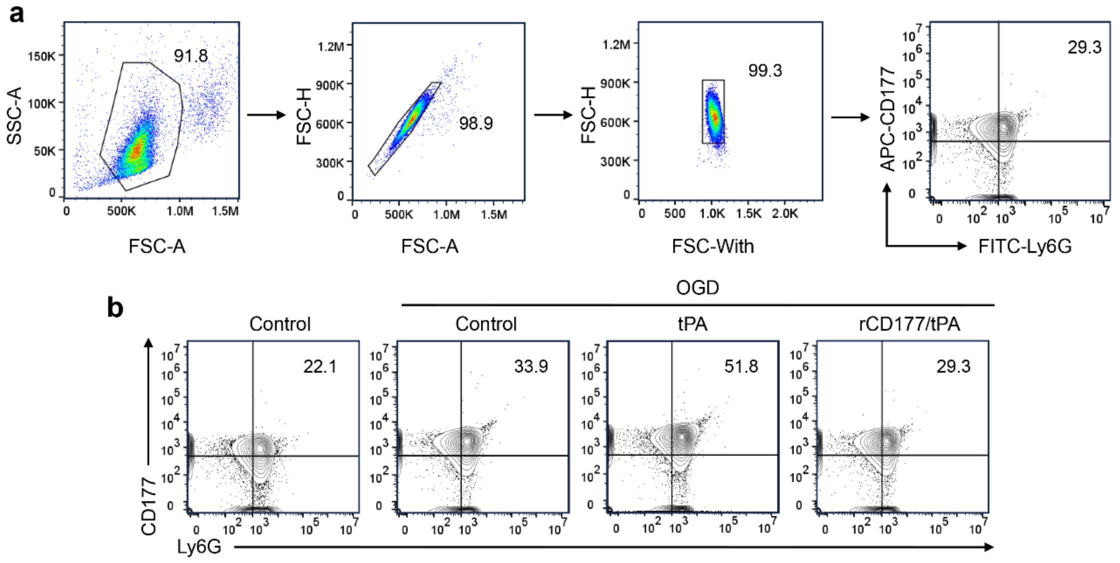

Supplementary Fig. 24. Expression of CD177$^+$LY6G$^+$ cells in migrated cells of the lower chamber. a. Flow cytometry gating strategy for identifying CD177$^+$Ly6G$^+$ cells in the migrated neutrophils. **b.** Representative flow cytometry plot of CD177$^+$Ly6G$^+$ cells in the lower chamber.

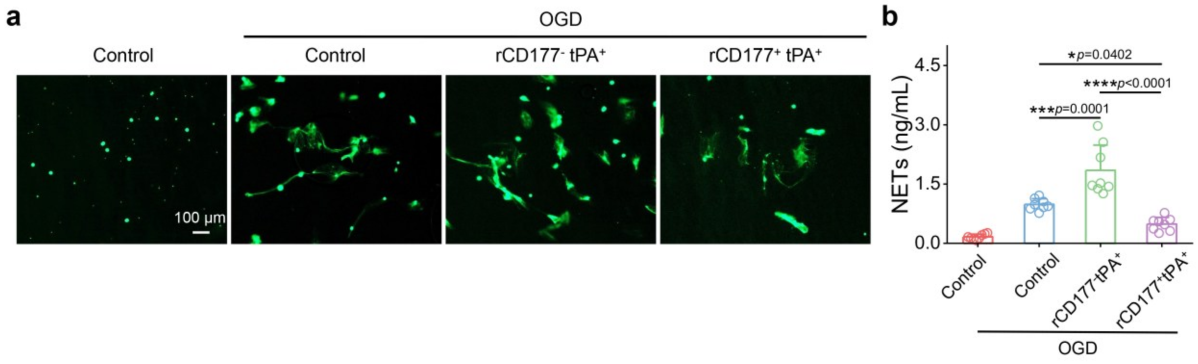

Supplementary Fig. 25. Expression of NETs in the lower chamber. a, Representative fluorescence images of NETs (SYTOX$^+$, green) in the lower chamber. **b,** The NETs levels in the lower chamber measured by ELISA (n = 12 biologically independent samples). Data are

presented as the mean ± SD. P values were calculated by one-way ANOVA followed by Bonferroni's post-hoc test. Source data are provided as a Source Data file.

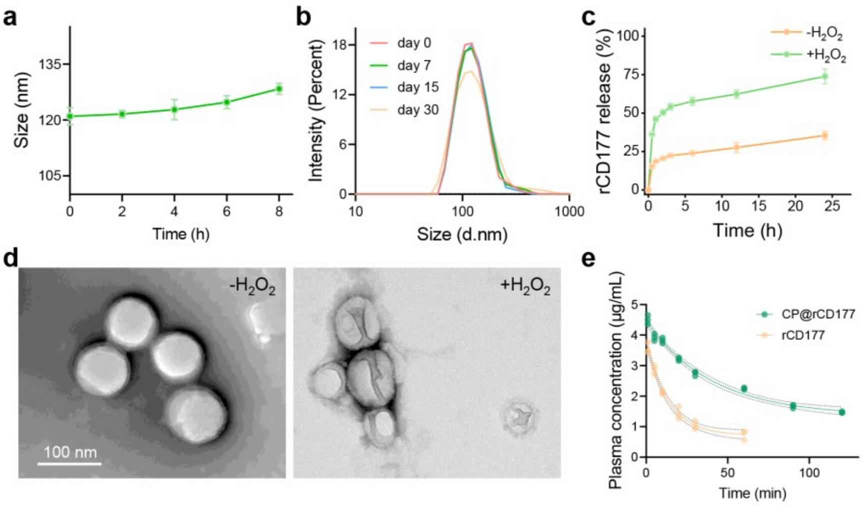

Supplementary Fig. 26. Characterization of CP@rCD177. a, Colloidal stability of CP@rCD177 incubated in FBS (pH 7.4) within 8 h at 37 °C. **b,** Size distribution of CP@rCD177 in PBS for 30 days. **c,** *In vitro* release of rCD177 from polymersomes in PBS or H_2O_2. **d,** Transmission electron microscopy (TEM) images of CP@rCD177 polymersomes in PBS or H_2O_2. **e,** In vivo pharmacokinetic profiles of CP@rCD177 and rCD177. The results in (**d**) were representative of three independent experiments. Data in (**a**, **c**, **e**) were presented as mean ± SD, $n = 3$ biologically independent samples. Source data are provided as a Source Data file.

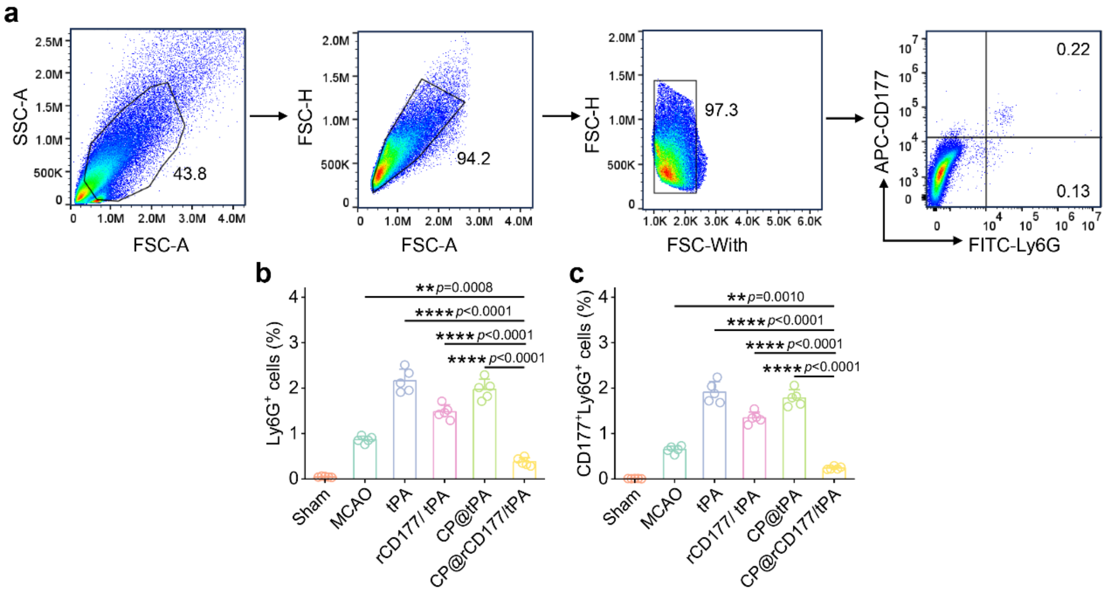

Supplementary Fig. 27. Expression of CD177⁺Ly6G⁺ cells in brain tissue of different treatment groups. a, Flow cytometry gating strategy for identifying CD177⁺Ly6G⁺ cells in mouse brains. **b**, Percentages of Ly6G⁺ cells (**a**) and CD177⁺Ly6G⁺ cells (**b**) according to flow cytometry analysis (n = 5 biologically independent samples). Data are presented as the mean ± SD. P values were calculated by one-way ANOVA followed by Tukey's post-hoc test. Source data are provided as a Source Data file.

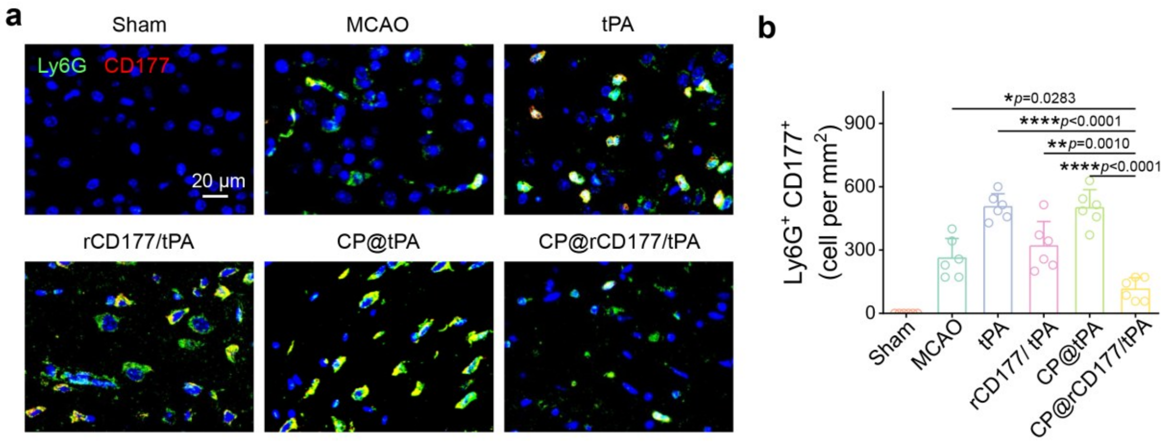

Supplementary Fig. 28. Expression of CD177$^+$Ly6G$^+$ cells in brain tissue of different treatment groups. a, Representative immunofluorescence staining images showing CD177-expressed neutrophils (red) in mice brain. Neutrophil were labeled with Ly6G (green), and the nuclei were stained with DAPI (blue). **b,** Quantitative CD177$^+$Ly6G$^+$ cells in mice brain based on immunofluorescence images (*n* = 6 biologically independent samples). Data are presented as the mean ± SD. *P* values were calculated by one-way ANOVA followed by Tukey's post-hoc test. Source data are provided as a Source Data file.

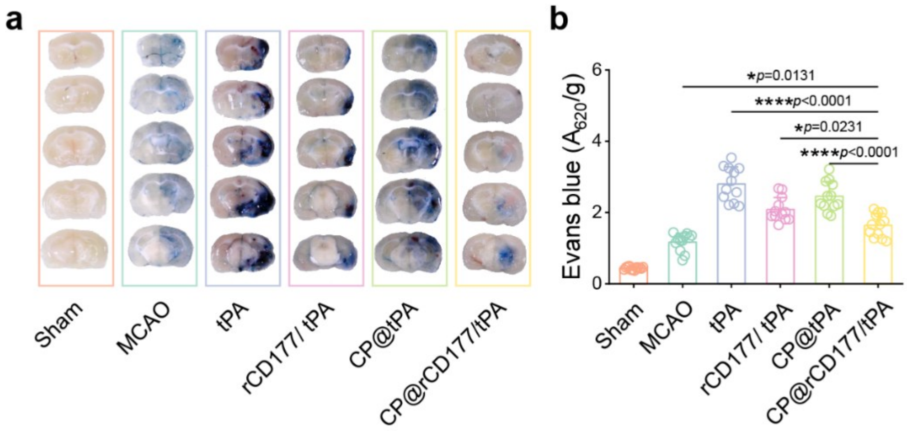

Supplementary Fig. 29. Blood-brain barrier (BBB) integrity at 22 h post-treatment. a, Digital photos showing Evans blue extravasation from the damaged BBB 22 h after treatment. **b,** Quantitative analysis of Evans blue (EB) content in different groups (*n* = 12 biologically independent samples). Data are presented as the mean ± SD. *P* values were calculated by one-way ANOVA followed by Tukey's post-hoc test. Source data are provided as a Source Data file.

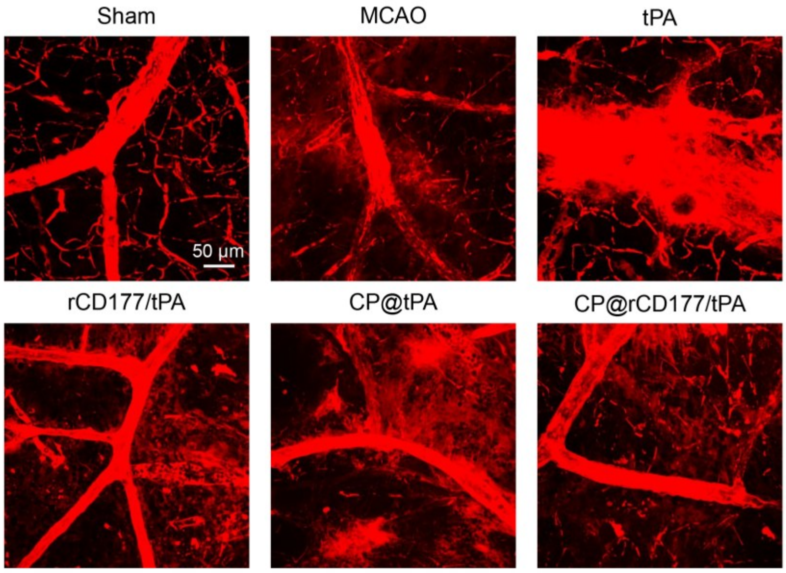

Supplementary Fig. 30. Intravital microscopy images of intravenously injected EB leakage in the cortical vessels at 22 h post-treatment. The results were representative of three independent experiments.

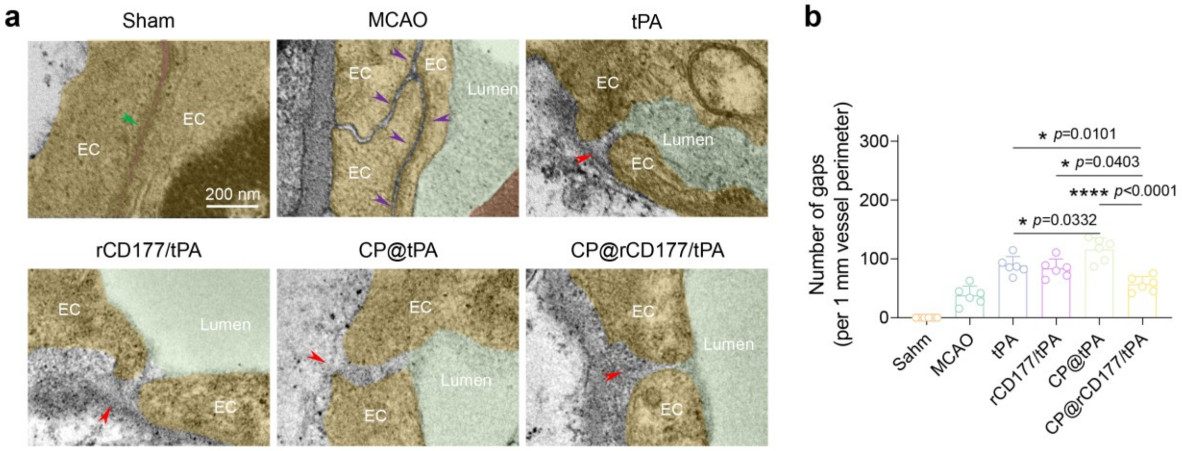

Supplementary Fig. 31. Observation and quantitative analysis of BBB integrity by TEM. **a**, TEM images illustrate the ultrastructure of the BBB in each treatment group. Green arrow, intact tight junction; purple arrow, open tight junction; red arrow, inter-endothelial gap. **b**, Number of gaps per 1 mm vessel perimeter in each treatment group (n = 6 biologically

independent samples). Data are presented as the mean ± SD. *P* values were calculated by one-way ANOVA followed by Tukey's post-hoc test. Source data are provided as a Source Data file.

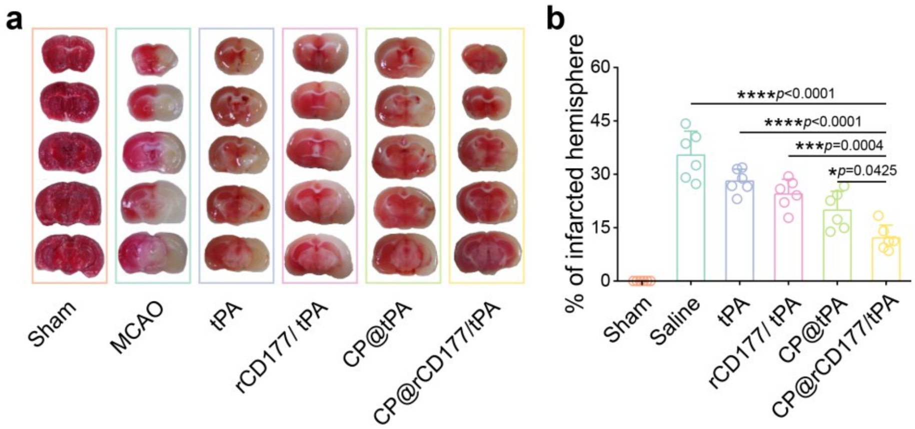

Supplementary Fig. 32. Detection and quantitative analysis of brain tissue injury by Triphenyltetrazolium chloride (TTC) staining. TTC staining (**a**) and infarct ratio (**b**) of MCAO mice 22 h after treatment (n = 6 biologically independent samples). Data are presented as the mean ± SD. *P* values were calculated by one-way ANOVA followed by Tukey's post-hoc test. Source data are provided as a Source Data file.

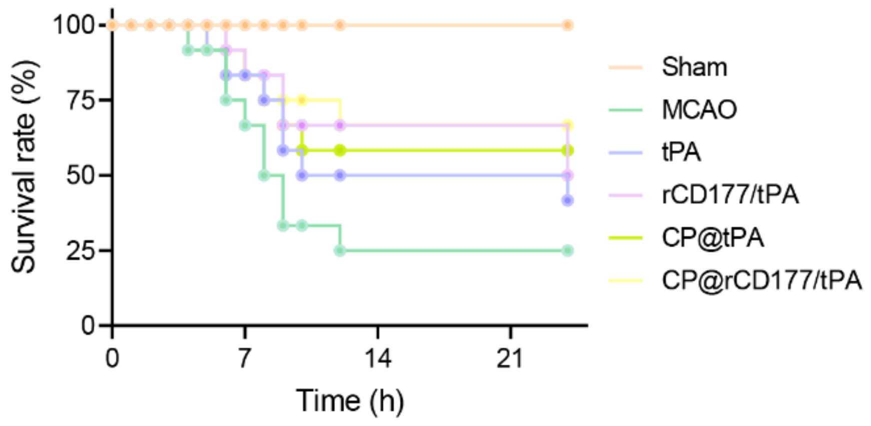

Supplementary Fig. 33. Survival rate in different treatment groups (*n* = 12 biologically independent samples. Source data are provided as a Source Data file.

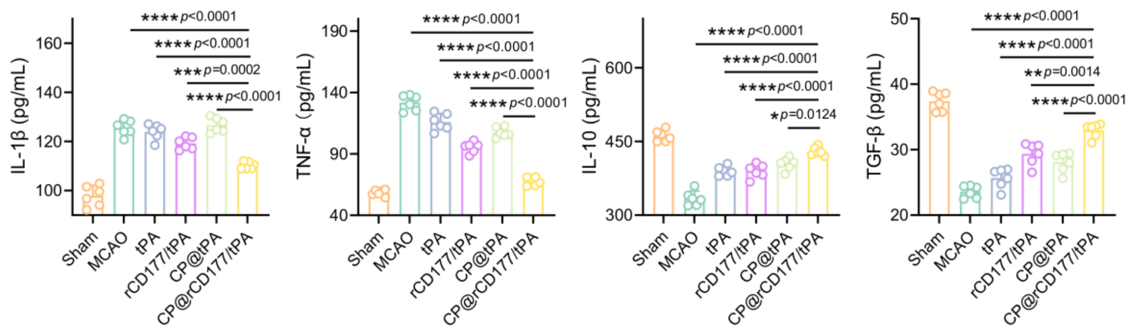

Supplementary Fig. 34. Evaluation of brain tissue inflammation after stroke. Expression levels of pro-inflammatory cytokines (IL-1β, TNF-α) and anti-inflammatory cytokine (IL-10, TGF-β) in the ischemic brains of MCAO mice on day 3 poststroke (*n* = 6 biologically independent samples). Data are presented as the mean ± SD. *P* values were calculated by one-way ANOVA followed by Tukey's post-hoc test. Source data are provided as a Source Data file.

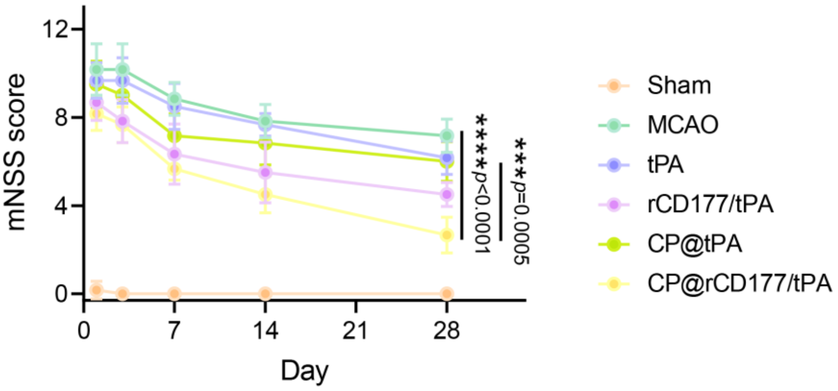

Supplementary Fig. 35. Neurological deficits assessed by the modified nneurological severity score (mNSS) (n = 6 biologically independent samples). Data are presented as the mean ± SD. P values were calculated by two-way ANOVA followed by Tukey's post-hoc test. Source data are provided as a Source Data file.

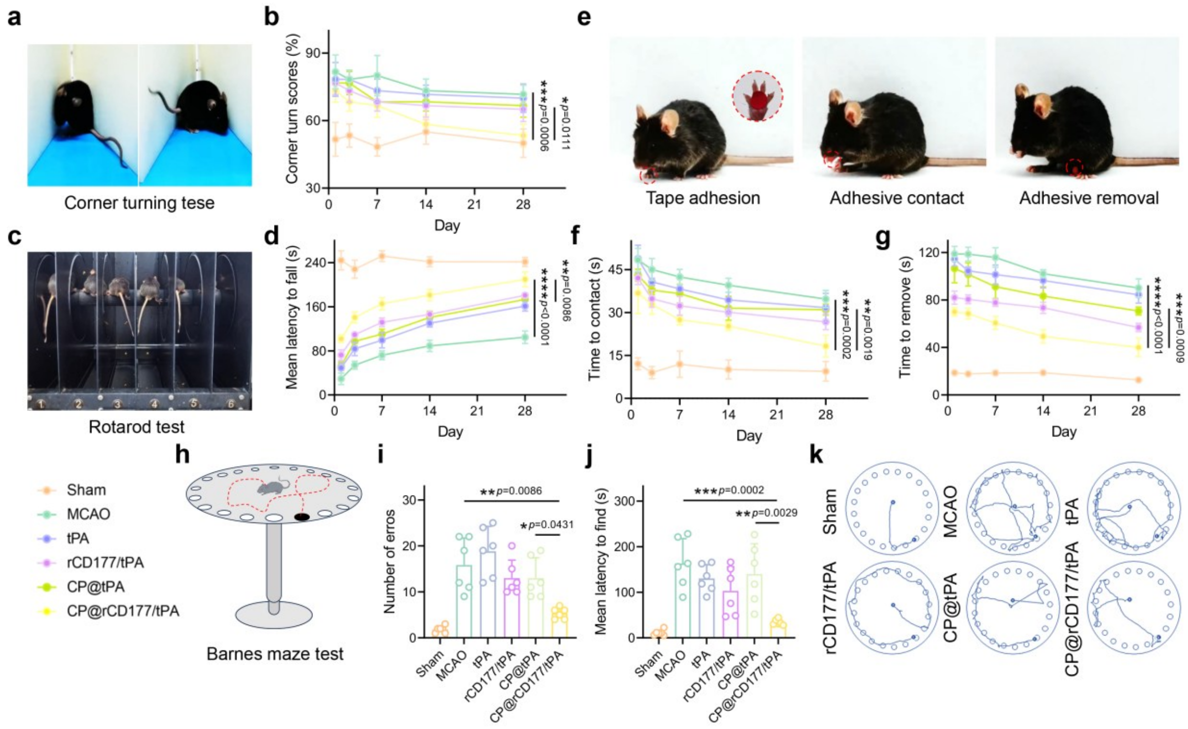

Supplementary Fig. 36. Long-term neurological recovery after stroke. a, Digital photo of the corner turn test. **b**, Laterality index measured in the corner turn test. **c**, Digital photo of the rotarod test. **d**, Mean latency to fall during the rotarod test. **e**, Digital photo of the adhesive test. **f**, Time to contact the tape in the adhesive test. **g**, Time to remove the tape in the adhesive test. **h**, Schematic of the Barnes maze test. **i**, Number of errors (**i**) and latency to find the escape hole (**j**) in the Barnes maze test. **k**, Representative images of walking path in different groups at day 28 after MCAO. Data in (**b**, **d**, **f**, **g**, **i**, **j**) were presented as mean ± SD, *n* = 6 biologically independent samples. *P* values were calculated by one-way or two-way ANOVA followed by Tukey's post-hoc test. Source data are provided as a Source Data file.

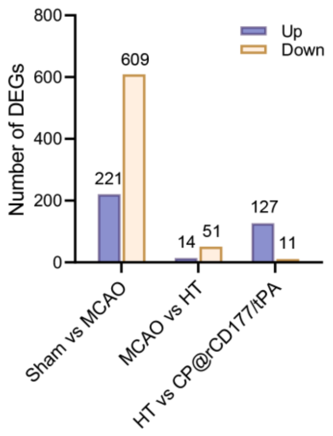

Supplementary Fig. 37. Differences in gene expression changes between various treatment groups. Source data are provided as a Source Data file.

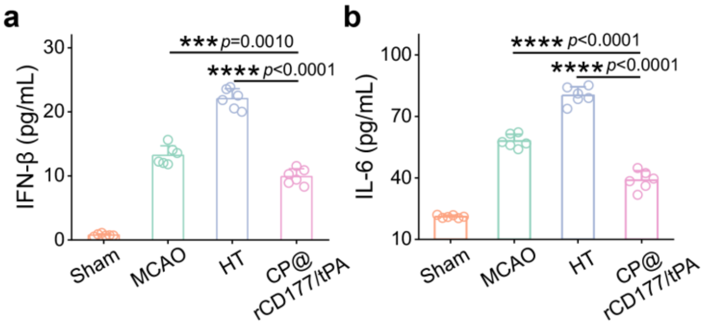

Supplementary Fig. 38. Detection of inflammatory factors. a,b, The IL-6 and IFN-β levels in mouse brains measured by enzyme-linked immunosorbent assay (ELISA) at 22 h post-treatment. Data in (**a**, **b**) were presented as mean ± SD, $n = 6$ biologically independent samples. *P* values were calculated by one-way ANOVA followed by Bonferroni's post-hoc test. Source data are provided as a Source Data file.

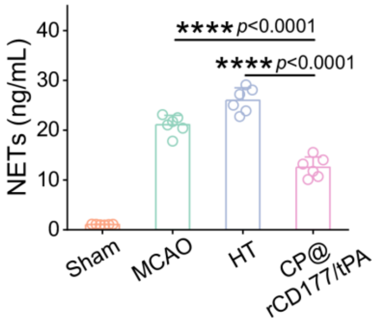

Supplementary Fig. 39. The NETs level in mouse brains measured by ELISA at 22 h post-treatment ($n = 6$ biologically independent samples). Data are presented as the mean ± SD. *P* values were calculated by one-way ANOVA followed by Bonferroni's post-hoc test. Source data are provided as a Source Data file.

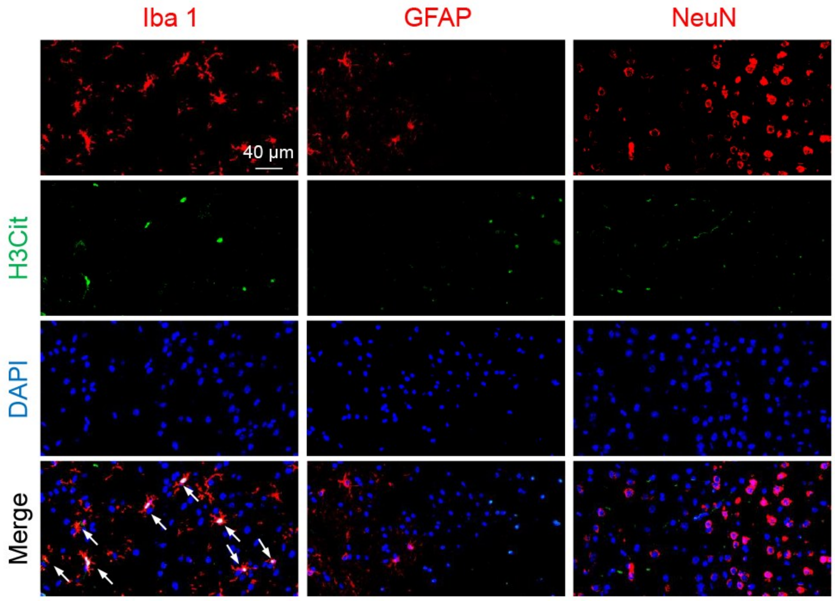

Supplementary Fig. 40. Representative immunofluorescence staining images showing the colocalization of NETs (H3Cit^{+}, green) in microglia (Iba-1^{+}, red), astrocytes (GFAP^{+}, red), and neurons (NeuN^{+}, red) in the HT brains at 22 h post-treatment. White arrows indicate cells with NETs colocalization. The results were representative of three independent experiments.

2. Supplementary Tables

Supplementary Table 1. Characterization of different tPA polymersomes (n = 3 biologically independent samples).

Polymersomes	Size (nm)	PDI	Zeta potential (mV)	DL (%)	EE (%)
CP@tPA	125.7±6.51	0.157±0.017	-3.43±0.74	7.55±0.44	81.73±5.12
P@tPA	127.67±3.33	0.197±0.019	-9.52±0.76	7.47±0.49	80.75±5.78
Null@tPA	138.13±6.72	0.163±0.040	-6.16±0.27	7.25±0.14	78.12±1.59

Supplementary Table 2. Pharmacokinetic parameters of free tPA and CP@tPA (*n* = 3 biologically independent samples).

Sample	$t_{1/2}$ (min)	Ke (1/min)	V1 (L/kg)	CL (mL/min/kg)	AUC (0-t) (mg/L*min)
tPA	6.986±1.535	0.103±0.025	0.197±0.016	20.033±3.556	45.984±8.401
CP@tPA	65.747±3.750	0.010±0.001	0.217±0.010	2.285±0.069	287.025±10.846

Supplementary Table 3. Characterization of CP@rCD177 polymersomes (*n* = 3 biologically independent samples).

Polymersomes	Size (nm)	PDI	Zeta potential (mV)	DL (%)	EE (%)
CP@rCD177	124.3±2.77	0.183±0.026	-3.85±0.75	6.62±0.13	72.82±1.39

Supplementary Table 4. Pharmacokinetic parameters of rCD177 and CP@rCD177 (*n* = 3 biologically independent samples).

Sample	$t_{1/2}$ (min)	Ke (1/min)	V1 (L/kg)	CL (mL/min/kg)	AUC (0-t) (mg/L*min)
rCD177	17.146±2.366	0.041±0.006	0.248±0.020	10.103±1.061	83.375±3.577
CP@rCD177	66.602±2.319	0.010±0.001	0.212±0.005	2.208±0.094	291.585±1.320

Supplementary Table 5. Antibodies used for this work.

Antibodies	Provider	Catalog	Dilution
Ly6G	Thermo Fisher scientific	11-9668-80	1:1000
CD177	BD	566599	1:50
Ly6G	BioLegend)	127607	1:100
Rat anti-Ly6G antibody	Abcam	ab25377	1:100
rabbit anti-CD177 antibody	Servicebio	GB11316-[illegible]	1:1000
Alexa Fluor 488-conjugated goat anti-rat IgG	Abcam	ab150157	1:1000
Alexa Fluor 594-conjugated donkey anti-rabbit IgG	Abcam	ab150076	1:1000
rat anti-CD31	Abcam	ab256569	1:1000
Anti-Fibrinogen	Abcam	Ab92572	1:1000
Alexa Fluor 647-conjugated goat anti- rat IgG	Abcam	ab150159	1:1000
H3cit	Abcam	ab219407	1:1000
NeuN	Abcam	ab279297	1:100
IBA-1	Abcam	ab283346	1:100
GFAP	Abcam	ab279291	1:200
CD62P	Biolegend	148303	
GADD45	Thermo Fisher scientific	PA5-43160	1:1000
TSP1	Solarbio	K007665P	1:1000
COX2	Solarbio	K009752P	1:1000
ANGPT2	Solarbio	K001733P	1:1000
β-Actin	Cell Signaling Technology	4967S	1:1000
