# Supplementary material for: Polymersomes preventing brain infiltration of CD177+ neutrophils to mitigate hemorrhagic transformation post-tPA thrombolysis
Source: Nat Commun. 2026 Mar 25;17:4395. doi: 10.1038/s41467-026-71076-w (PMC13181025; doi:10.1038/s41467-026-71076-w)
Supplement: Supplementary file 2 — Reporting Summary [file 41467_2026_71076_MOESM2_ESM.pdf]

nature portfolio

Corresponding author(s): Xing Guo

Last updated by author(s): Mar 10, 2026

Reporting Summary

Nature Portfolio wishes to improve the reproducibility of the work that we publish. This form provides structure for consistency and transparency in reporting. For further information on Nature Portfolio policies, see our Editorial Policies and the Editorial Policy Checklist.

Statistics

For all statistical analyses, confirm that the following items are present in the figure legend, table legend, main text, or Methods section.

n/a	Confirmed	
☐	☒	The exact sample size (*n*) for each experimental group/condition, given as a discrete number and unit of measurement
☐	☒	A statement on whether measurements were taken from distinct samples or whether the same sample was measured repeatedly
☐	☒	The statistical test(s) used AND whether they are one- or two-sided<br>*Only common tests should be described solely by name; describe more complex techniques in the Methods section.*
☒	☐	A description of all covariates tested
☐	☒	A description of any assumptions or corrections, such as tests of normality and adjustment for multiple comparisons
☐	☒	A full description of the statistical parameters including central tendency (e.g. means) or other basic estimates (e.g. regression coefficient) AND variation (e.g. standard deviation) or associated estimates of uncertainty (e.g. confidence intervals)
☐	☒	For null hypothesis testing, the test statistic (e.g. *F*, *t*, *r*) with confidence intervals, effect sizes, degrees of freedom and *P* value noted<br>*Give P values as exact values whenever suitable.*
☒	☐	For Bayesian analysis, information on the choice of priors and Markov chain Monte Carlo settings
☒	☐	For hierarchical and complex designs, identification of the appropriate level for tests and full reporting of outcomes
☒	☐	Estimates of effect sizes (e.g. Cohen's *d*, Pearson's *r*), indicating how they were calculated

Our web collection on statistics for biologists contains articles on many of the points above.

Software and code

Policy information about availability of computer code

Data collection	1H NMR (AM 300, Bruker, Germany); Malvern Zetasizer Nano ZS90(Malvern, UK); Transmission electron microscopy (TEM, TECNAI G2F20FEI, USA); In vivo imaging system (AniView Phoenix full-spectrum animal imaging system, China); Confocal microscope (A1R+, Nikon, Japan); RFLSI III Laser Speckle Blood Flow Imaging System (RFLSI III., RWD); Digital Microscope (DOM-1001, RWD); Scanning electron microscope (SEM, JSM-IT700HR, Japan); Two-photon microscopy (A1RMP＋, Nikon, Japan); CytoFLEX(Beckman, USA); Fluorescence microscope (Axio observer Z1, Zeiss, Germany); IntraVital Microscopy (IVM-CMS-3, Shanghai Aifei Electronic Technology Co., Ltd.); Biorad Gene Pulser Xcell (USA); Photoacoustic Multimode Small Animal In Vivo Imaging System (GAni-Plus, Guangzhou G-Cell Technology Co., Ltd.); Fluorescence spectrophotometer (Hitach F-7000, Japan);Microplate reader (Elx800, BioTek, USA); SMART 3.0 behavioral recording system (Panlab, Spain); Illumina Novaseq Xplus (USA);Rota Rod System (LE8200, RWD)。
Data analysis	MestReNova (version 14.0.0-23239); Zetasize software (version 8.01.4906); FlowJo (version 10.8.1); ImageJ software (version 2.0.0); Phoenix (version 100.0001); NIS-Element viewer (version 5.21); Zen (version 3.4); Origin (version 2024); GraphPad Prism (version 9.5.0); Imaris Viewer10.0.1; Case Viewer (version 2.4); IVM Studio (version 2312.040); Olympus OlyVia (version 4.1); Multimodal Photoacoustic Imaging System Software (version 2.0);Laser Speckle Blood Flow Imaging System (version 4.0); DAS (version 2.0); DESeq2 (v1.42.0)

For manuscripts utilizing custom algorithms or software that are central to the research but not yet described in published literature, software must be made available to editors and reviewers. We strongly encourage code deposition in a community repository (e.g. GitHub). See the Nature Portfolio guidelines for submitting code & software for further information.

Data

Policy information about availability of data

All manuscripts must include a data availability statement. This statement should provide the following information, where applicable:

- Accession codes, unique identifiers, or web links for publicly available datasets
- A description of any restrictions on data availability
- For clinical datasets or third party data, please ensure that the statement adheres to our policy

All data supporting the results of this study are included in the article and Supplementary Information. Source data are provided with this paper.

Research involving human participants, their data, or biological material

Policy information about studies with human participants or human data. See also policy information about sex, gender (identity/presentation), and sexual orientation and race, ethnicity and racism.

Reporting on sex and gender	Patients were not stratified by sex.
Reporting on race, ethnicity, or other socially relevant groupings	Asian
Population characteristics	The patients were individuals with ischemic stroke who developed HT following thrombolytic therapy with tPA.
Recruitment	All patients were recruited randomly, and written informed consent was obtained from each patient.
Ethics oversight	Third People Hospital of Chengdu (2024-S-154).

Note that full information on the approval of the study protocol must also be provided in the manuscript.

Field-specific reporting

Please select the one below that is the best fit for your research. If you are not sure, read the appropriate sections before making your selection.

☒ Life sciences ☐ Behavioural & social sciences ☐ Ecological, evolutionary & environmental sciences

For a reference copy of the document with all sections, see nature.com/documents/nr-reporting-summary-flat.pdf

Life sciences study design

All studies must disclose on these points even when the disclosure is negative.

Sample size	Simple sizes of 3-12 biologically independent samples or animals per group were used for in vitro and in vitro studies, respectively, as indicated for specific experiments in figure legends. We adhered to sample size requirements necessary for determining statistical significance with reference to the numbers used in recent relevant publications.
Data exclusions	No data were excluded from the analyses.
Replication	All experiments have at least 3 biologically independent replicates, the number of which is indicated in figure legends.
Randomization	Mice are allocated randomly to each treatment group.
Blinding	During data analysis for the experiments in this manuscript, we were blinded to group allocation and randomly alocated into experimental groups.

Reporting for specific materials, systems and methods

We require information from authors about some types of materials, experimental systems and methods used in many studies. Here, indicate whether each material, system or method listed is relevant to your study. If you are not sure if a list item applies to your research, read the appropriate section before selecting a response.

Materials & experimental systems

n/a	Involved in the study
	☒ Antibodies
	☒ Eukaryotic cell lines
☒	☐ Palaeontology and archaeology
	☒ Animals and other organisms
	☒ Clinical data
☒	☐ Dual use research of concern
☒	☐ Plants

Methods

n/a	Involved in the study
☒	☐ ChIP-seq
	☒ Flow cytometry
☒	☐ MRI-based neuroimaging

Antibodies

Antibodies used

Ly6G (1:100 for Flow, 11-9668-80, Thermo Fisher scientific)
CD177 (1:50 for Flow, 566599, BD)
Ly6G (1:100 for Flow, 127607, BioLegend)
Rat anti-Ly6G antibody (1:100 for IF, ab25377, Abcam)
rabbit anti-CD177 antibody (1:1000 for IF,GB11316-100,Servicebio)
Alexa Fluor 488-conjugated goat anti-rat IgG (1:1000 for IF, ab150157, Abcam);
Alexa Fluor 594-conjugated donkey anti-rabbit IgG (1:1000 for IF, ab150076, Abcam);
rat anti-CD31 (1:1000 for IF, ab256569, Abcam)
Anti-Fibrinogen (1:1000 for IF, ab92572, Abcam)
Alexa Fluor 647-conjugated goat anti- rat IgG (1:1000 for IF, ab150159, Abcam);
H3cit (1:800 for IF, 1:100 for IF, ab219407, Abcam);
NeuN (1:100, ab279297,Abcam)
IBA-1 (1:100 for IF, ab283346, Abcam);
GFAP (1:200 for IF, ab279291, Abcam);
CD62P (148303, Biolegend).
GADD45(1:1000 for WB, PA5-43160, Thermo Fisher scientific)
TSP1(1:1000 for WB, K007665P, Solarbio)
COX2(1:1000 for WB, K009752P, Solarbio)
ANGPT2(1:1000 for WB, K001733P, Solarbio)
β-Actin (1:1000 for WB, 4967S, Cell Signaling Technology)

Validation

The antibodies used in the study were all purchased from reputable commercial sources. All antibodies are widely used and validated by the providers or previous publications. Below are the manufacturer's links to the antibody information and relevant citations.
LY6G (11-9668-80, Thermo Fisher scientific) (https://www.thermofisher.cn/cn/zh/antibody/product/Ly-6G-Antibody-clone-1A8-Ly6g-Monoclonal/11-9668-80);
CD177 (566599, BD) (https://www.bdbiosciences.com/en-lu/products/reagents/flow-cytometry-reagents/research-reagents/single-color-antibodies-ruo/alexa-fluor-647-rat-anti-mouse-cd177.566599?tab=product_details);
Ly6G (127607, BioLegend)) (https://www.biolegend.com/en-ie/products/pe-anti-mouse-ly-6g-antibody-4777)
Ly6G (ab25377, Abcam)(https://www.abcam.cn/ly6g--ly6c-antibody-rb6-8c5-ab25377.html);
CD177(GB11316-100,Servicebio) (https://www.servicebio.cn/goodsdetail?id=1143);
Alexa Fluor 488-conjugated goat anti-rat IgG (ab150157, Abcam)(https://www.abcam.cn/goat-rat-igg-hl-alexa-fluor-488-ab150157.html);
Alexa Fluor 594-conjugated donkey anti-rabbit IgG (ab150076, Abcam) (https://www.abcam.cn/products/secondary-antibodies/donkey-rabbit-igg-hl-alexa-fluor-594-ab150076.html);
rat anti-CD31 (ab256569, Abcam) (https://www.abcam.cn/products/primary-antibodies/cd31-antibody-mec-133-ab256569.html);
Anti-Fibrinogen (ab92572, Abcam) (https://www.abcam.cn/products/primary-antibodies/fibrinogen-alpha-chain-antibody-epr2919-ab92572)
Alexa Fluor 647-conjugated goat anti- rat IgG (ab150159, Abcam) (https://www.abcam.cn/products/secondary-antibodies/goat-rat-igg-hl-alexa-fluor-647-ab150159.html);
H3cit (ab219407, Abcam)(https://www.abcam.cn/histone-h3-citrulline-r17-antibody-epr20358-120-ab219407.html);
Neun (ab279297, Abcam) (https://www.abcam.cn/products/primary-antibodies/neun-antibody-epr12763-rat-igg2a-chimeric-ab279297.html)
IBA-1 (ab283346, Abcam)(https://www.abcam.cn/products/primary-antibodies/iba1-antibody-epr16589-rat-igg2a-chimeric-ab283346.html);
GFAP (ab279291, Abcam)(https://www.abcam.cn/products/primary-antibodies/gfap-antibody-epr1034y-rat-igg2a-chimeric-ab279291.html);
CD62P (148303, biolegend) (https://www.biolegend.com/en-us/products/apc-anti-mouse-rat-cd62p-p-selectin-antibody-10805);
GADD45(PA5-43160, Thermo Fisher scientific) (https://www.thermofisher.cn/cn/zh/antibody/product/GADD45B-Antibody-Polyclonal/PA5-43160);
TSP1(K007665P, Solarbio) (https://www.solarbio.com/goodsInfo?id=62981);
COX2(K009752P, Solarbio) (https://www.solarbio.com/goodsInfo?id=759);
ANGPT2(K001733P, Solarbio) (https://www.solarbio.com/goodsInfo?id=58404);
β-Actin (4967S, Cell Signaling Technology) (https://www.cellsignal.cn/products/primary-antibodies/b-actin-antibody/4967).

Eukaryotic cell lines

Policy information about cell lines and Sex and Gender in Research

Cell line source(s)	Mouse brain capillary endothelial bEnd.3 cells were obtained from iCell Bioscience Inc (Shanghai, China). Neutrophils were extracted from the blood of male C57BL/6J mice.
Authentication	bEbd.3 cell lines were used for less than 1 month since purchase and neutrophils for less than 24 hours from extraction.
Mycoplasma contamination	All the cell lines were tested termly and they were negative for mycoplasma contamination.
Commonly misidentified lines (See ICLAC register)	None of the cell lines used in this research are listed in the database of commonly misidentified cell lines.

Animals and other research organisms

Policy information about studies involving animals; ARRIVE guidelines recommended for reporting animal research, and Sex and Gender in Research

Laboratory animals	Male C57BL/6J mice (8-12 weeks, 28-32 g) were obtained from Beijing Huafukang Biotechnology Co., Ltd. The animals were hosted in SPF barrier environment at 25 ℃ with a 12h dark/light cycle and have access to food and water.
Wild animals	No wild animals were used in this study.
Reporting on sex	Male mice were used in this study.
Field-collected samples	No Field-collected samples used.
Ethics oversight	All animal experiments were conducted under protocols approved by the Institutional Animal Care and Use Committee of Southwest Jiaotong Universtiy.

Note that full information on the approval of the study protocol must also be provided in the manuscript.

Clinical data

Policy information about clinical studies

All manuscripts should comply with the ICMJE guidelines for publication of clinical research and a completed CONSORT checklist must be included with all submissions.

Clinical trial registration	The study was approved by the Ethics Review Committee of the Third People Hospital of Chengdu (2024-S-154).
Study protocol	Collection of peripheral blood from stroke patients after unused drugs and tPA thrombolysis.
Data collection	The transcriptome analysis was performed by constructing the libraries and sequencing by Shanghai Majorbio Bio-Pharm Biotechnology Co., Ltd. (China).
Outcomes	The blood samples from stroke patients and those with HT were collected from the same individuals, and the blood samples passed the quality inspection, making them suitable for subsequent transcriptomic analysis.

Plants

Seed stocks	n/a
Novel plant genotypes	n/a
Authentication	n/a

Flow Cytometry

Plots

Confirm that:

- [x] The axis labels state the marker and fluorochrome used (e.g. CD4-FITC).
- [x] The axis scales are clearly visible. Include numbers along axes only for bottom left plot of group (a 'group' is an analysis of identical markers).
- [x] All plots are contour plots with outliers or pseudocolor plots.
- [x] A numerical value for number of cells or percentage (with statistics) is provided.

Methodology

Sample preparation	For blood samples, mouse blood was mixed with PBS and red blood cell sedimentation solution at a volume ratio of 1:1:1. The mixture was allowed to stand at room temperature for 30 minutes and then subjected to density gradient centrifugation to collect the supernatant and isolate neutrophils for further analysis. For brain tissue samples, the tissues were minced into small pieces and homogenized, followed by mechanical processing through a 40 µm cell strainer to generate a single cell suspension for analysis by flow cytometry.
Instrument	CytoFLEX(Beckman, USA)
Software	FlowJo (version 10.8.1)
Cell population abundance	The instrument counts 10000 to 50000 cells autonomously.
Gating strategy	Cells were gated by forward scattering height (FSC-H) and lateral scattering height (SSC-H). Neutrophils were labeled with FITC-Ly6G (1:100, Thermo Fisher scientific), APC-CD177 (1:50, BD Pharmingen).

- [x] Tick this box to confirm that a figure exemplifying the gating strategy is provided in the Supplementary Information.
